# 

**Published:** 1913-10-18

**Authors:** 


### Gifts and Bequests.

The London Hospital has received a cheque for £200 on behalf of Alexandra Day.

Mrs. Elizabeth Oakley, of Hove, has bequeathed £50 to the Lady Chichester Hospital, Hove.

A legacy of £500 has been received from the late Mr. J. T. Walton by the Manchester Children's Hospital.

By the death of Mr. Thomas Ryder, of Harrogate, a sum of £20,000 left by his wife to build and endow a hospital at Middleton, Lancs, now becomes available.

Mr. James Cloudsley, of Tottenham, has left nearly £4,000 for religious and charitable purposes, including £1,000 to the Prince of Wales's Hospital, Tottenham.

Mrs. Isabella Riddle Glover or Caird, who died in Edinburgh on September 8, widow of Principal John Caird, of Glasgow University, has bequeathed £1,000 to the Western Infirmary, Glasgow.

Mr. Christopher Taylor, of Brampton, Cumberland, formerly a chemist, who left £110,664, has bequeathed £4,000 to the Cumberland Infirmary, Carlisle; £2,000 to the Home and Sanatorium for Consumptives, Threlkeld, Cumberland; £1,000 to the Carlisle Home for Incurables and to the Silloth Convalescent Institution.

Mr. Joseph Carter Bell, of Higher Broughton, Salford, A.R.S.M., F.I.C., public analyst for Cheshire and for Salford, who left estate valued at £34,427 gross, has bequeathed of the residue of his estate three-sixtieths each to the Manchester Royal Infirmary, the Salford Royal Hospital, and the London Orphan Asylum, Watford.

Mrs. Emma James, of Eastbourne, has bequeathed £3,000 to Nottingham Hospital, in memory of her husband; £500 each to the Royal Hospital for Incurables, Putney, and the City of London Hospital for Diseases of the Chest; £1,000 each to the Princess Alice Memorial Hospital, Eastbourne, the Cancer Hospital, Brompton, the Prince of Wales's Hospital Fund (*sic*), and Muller's Orphanage, Bristol.

Mr. Joseph Staincliffe Hurst, of Ripon, Yorks, a prominent supporter of local hospitals, who died on June 11, aged eighty-six, has left estate in the United Kingdom the net personalty of which has been sworn at £263,284. The duties on the property at this valuation will amount to about £35,000. The testator has left £500 to the Mirfield Memorial Hospital, and £250 each to Ripon Dispensary and Cottage Hospital and the Ripon Victoria Nursing Institute.

### LARGE BEQUESTS TO LONDON HOSPITALS.

Mr. Charles Thomas Harris, senior member of the Court of Common Council, an alderman of the Camberwell Borough Council, has left in stock of the South Metropolitan Gas Company £5,000 each to the London Hospital and King's College Hospital; £2,000 to Lord Mayor Treloar Cripples' Home, Alton; and £1,000 each to the Infant Orphan Asylum, the London Orphan Asylum, the Metropolitan Convalescent Institution, and the Samaritan Fund of St. Thomas's Hospital. He also left £312 Three per Cent. North British Railway Debentures to the Camberwell Provident Dispensary; and his collection of pictures and two Japanese gilt bronze cloisonné bowls upon trust for sale at Christie's when his executors may choose, directing them meanwhile to have the items fully catalogued and details freely sent to those taking an interest in hospitals and hospital work, the proceeds of such sale after payment of expenses to be given to King's College Hospital Removal Fund. The testator left considerable bequests to relatives and others, and the residue of his property to St. Thomas's Hospital.

